# RETRACTION: Effect of a Neural Relay on Liver Regeneration in Mice: Activation of Serotonin Release from the Gastrointestinal Tract

**DOI:** 10.1002/2211-5463.13961

**Published:** 2024-12-25

## Abstract

**RETRACTION:** R. Inoue, K. Kamimura, T. Nagoya, N. Sakai, T. Yokoo, R. Goto, K. Ogawa, Y. Shinagawa‐Kobayashi, Y. Watanabe‐Mori, A. Sakamaki, S. Abe, H. Kamimura, N. Miyamura, H. Nishina, and S. Terai, “Effect of a Neural Relay on Liver Regeneration in Mice: Activation of Serotonin Release from the Gastrointestinal Tract,” *FEBS Open Bio* 8, no. 3 (2018): 449‐460, https://doi.org/10.1002/2211‐5463.12382.

The above article, published online on 16 January 2018, in Wiley Online Library (http://onlinelibrary.wiley.com/),has been retracted by agreement between the journal Editor‐in‐Chief, Miguel A. De la Rosa; FEBS Press; and John Wiley and Sons Ltd. The journal received a report from a third party which indicated that the Day 2 and Day 4 panels in Figure 3A had been duplicated and rotated. Additional investigation by the journal discovered multiple inappropriate image duplications and overlaps in Figure 6A. The authors responded to an inquiry by the journal, confirmed that images had been duplicated in both figures, and provided what was labelled as the correct data. Following receipt of the authors' explanation and new data, the journal requested an investigation by the authors' institution.

The institutional investigation reported that there was insufficient evidence of intentional image manipulation and concluded that the image duplications were due to errors in image preparation by the authors. However, given the extent of the identified issues, the editors have lost confidence in the data presented and consider the conclusions of this manuscript substantially compromised. As a result, the Editor‐in‐Chief, FEBS Press, and John Wiley and Sons Ltd. have determined that a retraction is necessary. The authors disagree with the retraction.

**RETRACTION:** R. Inoue, K. Kamimura, T. Nagoya, N. Sakai, T. Yokoo, R. Goto, K. Ogawa, Y. Shinagawa‐Kobayashi, Y. Watanabe‐Mori, A. Sakamaki, S. Abe, H. Kamimura, N. Miyamura, H. Nishina, and S. Terai, “Effect of a Neural Relay on Liver Regeneration in Mice: Activation of Serotonin Release from the Gastrointestinal Tract,” *FEBS Open Bio* 8, no. 3 (2018): 449‐460, https://doi.org/10.1002/2211‐5463.12382.

The above article, published online on 16 January 2018, in Wiley Online Library (http://onlinelibrary.wiley.com/),has been retracted by agreement between the journal Editor‐in‐Chief, Miguel A. De la Rosa; FEBS Press; and John Wiley and Sons Ltd. The journal received a report from a third party which indicated that the Day 2 and Day 4 panels in Figure 3A had been duplicated and rotated. Additional investigation by the journal discovered multiple inappropriate image duplications and overlaps in Figure 6A. The authors responded to an inquiry by the journal, confirmed that images had been duplicated in both figures, and provided what was labelled as the correct data. Following receipt of the authors' explanation and new data, the journal requested an investigation by the authors' institution.

The institutional investigation reported that there was insufficient evidence of intentional image manipulation and concluded that the image duplications were due to errors in image preparation by the authors. However, given the extent of the identified issues, the editors have lost confidence in the data presented and consider the conclusions of this manuscript substantially compromised. As a result, the Editor‐in‐Chief, FEBS Press, and John Wiley and Sons Ltd. have determined that a retraction is necessary. The authors disagree with the retraction.

